# Maternal Adiponectin Decreases Placenta Nutrient Transport in Mice

**DOI:** 10.1096/fj.202403251RR

**Published:** 2025-04-18

**Authors:** Manisha Samad, Benjamin Ulfenborg, Sahar Soleimani Sani, Marco Bauzá Thorbrügge, Man Mohan Shrestha, Claes Ohlsson, Manuel Maliqueo, Elisabet Stener‐Victorin, Ingrid Wernstedt Asterholm, Anna Benrick

**Affiliations:** ^1^ Department of Physiology, Institute of Neuroscience and Physiology, Sahlgrenska Academy University of Gothenburg Gothenburg Sweden; ^2^ School of Bioscience University of Skövde Skövde Sweden; ^3^ Department of Internal Medicine and Clinical Nutrition, Institute of Medicine, Sahlgrenska Academy University of Gothenburg Gothenburg Sweden; ^4^ Laboratory of Endocrinology and Metabolism, Department of Internal Medicine West Division University of Chile Santiago Chile; ^5^ Department of Physiology and Pharmacology Karolinska Institute Stockholm Sweden; ^6^ School of Health Sciences University of Skövde Skövde Sweden

**Keywords:** adiponectin, amino acid transporters, fetal development, mice, obesity, placenta, pregnancy

## Abstract

Women with obesity who develop gestational diabetes have lower serum adiponectin throughout pregnancy, suggesting that low levels impair the ability to handle metabolic challenges during pregnancy. The placenta expresses adiponectin receptors, and adiponectin could therefore indirectly affect the developing fetus. Here, we aimed to investigate how elevated maternal and fetal adiponectin affect placental function, fetal growth, and metabolism during pregnancy in normal‐weight and obese mice. Wild‐type (wt) and adiponectin‐overexpressing (APNtg) mice were fed normal chow or a high fat/high sucrose (HF/HS) diet 8 weeks before and during pregnancy to induce obesity. Mice were euthanized and dissected on gestational day 18.5. Lipid, glucose, and amino acid tracers were administered to the obese pregnant dams to study nutrient uptake. The effects of elevated adiponectin on fetal liver and placental function were further investigated using global proteomics. A 40%–50% increase in serum adiponectin reduced fetal growth in dams fed a HF/HS diet, but not a normal chow diet. The uptake of glucose, lipid, and amino acid tracer was lower, along with decreased expression of several amino acid transporters in the placenta of APNtg dams on HF/HS diet. This suggests that adiponectin decreases placental transfer of nutrients. Livers of fetuses from APNtg dams showed downregulated lipid and amino acid metabolic pathways possibly reflecting an energy deficit. In conclusion, elevated serum adiponectin in obese dams reduced the placental transfer of nutrients, resulting in fetal growth restriction and altered fetal liver function. Maternal adiponectin levels were the main driver of placenta function. While this could be beneficial for pregnancy‐related complications like babies born large for their gestation age, our study indicates that adiponectin should be in an optimal concentration range, neither too low nor too high, to prevent these complications.

## Graphical abstract

The schematic illustrates the impact of adiponectin in placental function and fetal growth in obese pregnant mice. Adiponectin influences the placenta, by phosphorylation of mTOR at Ser2448 which alters its activity. This results in reduced nutrient influx (red arrows) to the fetus, ultimately contributing to fetal growth restriction. Created with BioRender.com

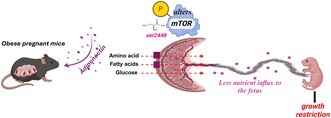

## 1 Introduction

According to the Barker theory and the concept of Developmental Origins of Health and Disease (DOHaD), metabolic diseases in adulthood may have their origins in intrauterine life [1, 2]. Maternal obesity and gestational diabetes are factors that can program the fetus to develop metabolic, reproductive, and behavioral disorders in adulthood [2, 3]. However, it is unclear whether direct fetal exposure causes these disorders or whether it is mediated by changes in placental function. Women with obesity during pregnancy and gestational diabetes are at increased risk of pregnancy complications and frequently deliver babies born large for gestational age [4]. Elevated blood glucose is associated with an increased risk of giving birth to large for gestational age babies as concurrent hyperinsulinemia plays a key role in stimulating prenatal growth [5]. Birth weight is a sensitive marker of the overall impact of the intrauterine environment and predicts the risk of developing metabolic syndrome in adulthood [6].

The adipocyte‐produced hormone adiponectin improves whole‐body metabolism [7]; low serum adiponectin is associated with insulin resistance, gestational diabetes, and obesity [8]. Maternal serum adiponectin peaks pregestation and gradually decreases as pregnancy progresses in both humans and mice, but women with obesity start and remain at lower levels compared to those of normal weight [8, 9]. They also show smaller changes in adiponectin levels [10], possibly indicating metabolic inflexibility [7]. Among women with obesity, those who develop gestational diabetes have lower serum adiponectin throughout pregnancy than their euglycemic counterparts [11], suggesting that low serum adiponectin impairs the capacity to manage metabolic changes during pregnancy.

The widespread presence of adiponectin receptors in peripheral organs enables adiponectin to influence whole‐body metabolism, with growing evidence pointing to a direct role in reproductive tissues [12, 13]. The placenta expresses adiponectin receptors [12], and this fact raises the intriguing possibility that adiponectin may exert endocrine effects on the placenta and influence fetal development. A significant drop in serum adiponectin during pregnancy is linked to large‐for‐gestational‐age babies [14], and deleting adiponectin in mice increases fetal growth in some [15, 16] but not all studies [17, 18]. Elevated serum adiponectin, on the other hand, may reduce weight gain in mice by reducing glucose and amino acid transport from the placenta to the fetus, and adiponectin supplementation may protect the fetus from the effects of maternal obesity [19, 20, 21]. The reduced glucose transport to the fetus is partly mediated by adiponectin's inhibitory effect on placental insulin signaling [19, 22], contrasting with adiponectin's insulin‐sensitizing effects in other peripheral organs.

Human and mouse fetuses produce adiponectin during the rapid growth phase in late gestation, coinciding with the formation and maturation of adipocytes [17, 18, 23]. Whether the placenta can synthesize adiponectin has been disputed, but the current state of knowledge shows that the placenta does not produce adiponectin [24]. Cord blood adiponectin is comparable to or even higher than maternal levels at birth, suggesting that fetal adiponectin peaks at birth in term pregnancies [23, 25]. Still, maternal and fetal adiponectin are not interchangeable since adiponectin is too large to pass through the placenta [17, 18], and the different origins likely play different roles in fetal development. However, it is not known whether fetal overexpression of adiponectin can affect placental function and fetal growth or how maternal adiponectin affects the fetus.

To answer these questions, we used transgenic mice overexpressing adiponectin (APNtg), with and without diet‐induced obesity. There is no difference in litter size between normal‐fed wild‐type and APNtg mothers, but the offspring of APNtg mothers are smaller at 3 weeks of age [26, 27]. This effect is partly due to poor milk quality in APNtg mothers, which impairs growth during lactation [26]. Whether the fetuses are already smaller before birth is unknown. Therefore, this study aimed to investigate how elevated maternal and fetal adiponectin levels affect placenta function, fetal growth, and metabolism during pregnancy in normal weight and obese mice, and if elevated serum adiponectin can protect against the effects of obesity on placenta function and fetal development.

## 2 Material and Methods

### 2.1 Mouse Model

The heterozygous adiponectin transgenic (APNtg) strain has a dominant mutation in the collagenous domain of adiponectin. One mutant allele is enough to overexpress adiponectin. The deletion mutant lacks 13 of 22 Gly‐X‐Y repeats in the domain, which improves the efficiency of adiponectin secretion and leads to a life‐long increase in circulating adiponectin [28]. Wild type (wt) and APNtg mice were genotyped as previously described [29]. Ear and tail biopsies from adult and fetal mice, respectively, were incubated at 55**°**C overnight in DirectPCR lysis buffer (Viagen #102‐T) and Proteinase K solution (Invitrogen Direct PCR #25530‐049, 0.2 mg/mL) and thereafter incubated at 85**°**C to inactivate the enzyme. The samples were centrifuged at 14 000× *g* for 3 min. Two microliters of each sample were mixed with 18 μL mastermix containing Qiagen HotstarTaq MMx (#1010023, Qiagen) and primers for genotyping APNtg mice; forward primers (5′‐GTTCCTCTTAATCCTGCCCAGTC‐3′) and reverse primers (5′‐CCCGGAATGTTGCAGTAGAACTTG‐3′) (0,5 μM) were used. The PCR reaction cycle was 5 min at 95°C, then 30 s at 95°C, 45 s at 64°C, and 1 min at 72°C for 30 cycles, followed by 8 min at 72°C. The PCR product was run on a 2% agarose gel in 1× TBE (45 mM Tis‐borate and 1 mM EDTA) containing Gel Nucleic Acid stain (#14G0326, Invitrogen). qPCR was performed using the GoTaq G2 Flexi DNA Polymerase kit (Promega) to determine the sex of the offspring. Each reaction was carried out in a total volume of 25 μL with a forward primer (5′‐GCCTCATCGGAGGGCTAAAG‐3′) (0.2 μM) and reverse primer (5′‐GTCCCACTGCAGAAGGTTGT‐3′) (0.2 μM). The mice were housed under a 12‐h light–dark cycle under standard conditions at the Laboratory of Experimental Biomedicine, University of Gothenburg, Sweden. Animals had *ad libitum* access to water and food, either a standard chow diet (CD; 7.42% fat, 17.49% protein and 75.09% carbohydrate; Special Diets Services, UK) or a hypercaloric high fat/high sucrose diet (HF/HS; 40% fat, 17% protein, and 43% carbohydrate [35% sucrose], 4.67 Kcal/g; D12079B Research Diets, Brogaarden, Denmark), in combination with 20% sucrose water (S9379, Sigma‐Aldrich) supplemented with Vitamin mix (10 g/4000 Kcal, V10001, Research Diets) and Mineral mix (35 g/4000 Kcal, S10001, Research Diets). Research Diets D12331; 58% fat, 17% protein, and 25% carbohydrate (18% sucrose) were used for the tracer experiment. Experiments were approved by the Animal Ethics Committee of the University of Gothenburg and were performed in accordance with the legal requirements of the European Community (Decree 86/609/EEC).

### 2.2 Experimental Design

**FIGURE 1:**
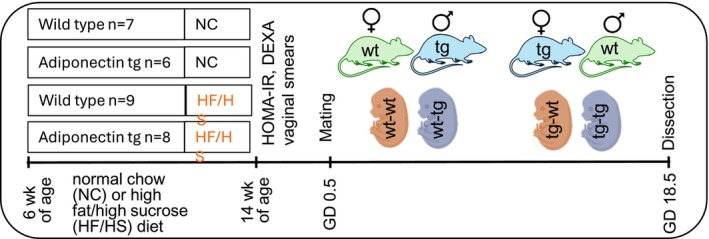
Study design for fetal development from adiponectin transgenic (APNtg) and wild‐type (wt) dams. Created with BioRender.com.

Wt and APNtg female mice were fed normal chow (wt *n* = 7, APNtg *n* = 6) or a HF/HS diet (wt *n* = 9, APNtg *n* = 8) for 8 weeks before mating (Figure 1). Females in the estrus phase, as determined by vaginal smears [30], were mated overnight with a male on normal chow. APNtg females were mated with wt males, and wt females were mated with APNtg males, generating wt and APNtg fetuses in both groups. The following morning was defined as gestational day (GD) 0.5, and body weights were recorded regularly to confirm pregnancy‐induced weight gain. Females continued the same diet after mating. On GD 18.5, dams were fasted for 4 h and anesthetized with 2.5% isoflurane inhalation (Isoba vet, Schering‐Plow, Uxbridge, UK). Axillary blood was collected for serum analyses; the dams were killed by heart removal, and organs, fetuses, and placentas were collected and weighed. The fetuses were sacrificed by cervical dislocation prior to dissection of livers. Tissues were snap frozen in liquid nitrogen and stored at −80°C until further analysis.

### 2.3 Dual‐Energy X‐Ray Absorptiometry

Bone, fat, and lean body mass were determined in females on normal chow or HF/HS diet before mating using the Lunar PIXImus Mouse Densitometer (Wipro GE Healthcare, Madison. USA). Mice were anesthetized during the procedure using isoflurane inhalation (2%; Isoba vet) [31].

### 2.4 In Vivo Tracer Placental Transport Studies

Two sets of experiments were conducted to determine the placental nutrient uptake from the mother to the fetus. The first experiment was to assess the lipid and glucose uptake in animals on the HF/HS diet (D12331, Research Diets, wt *n* = 4 dams and 28 placentas/fetuses, APNtg *n* = 5 dams and 32 placentas/fetuses). This was accomplished by employing [9,10‐3H(N)]‐triolein (2 μCi/mouse, Perkin Elmer, Boston, MA, USA) and D‐[U‐14C]‐glucose (5 μCi/mouse, Perkin Elmer, Boston, MA, USA) radio‐labeled tracers incorporated in an intralipid emulsion (20%, Sigma‐Aldrich, St Louis, MO, USA). The mice were fasted for 3.5 h before the experiment. An oral administration of 300 μL/mouse of radiolabeled tracer emulsion (glucose and lipid) was given. After 1 h, the mice were killed, and the tissues, namely, white adipose tissue (WAT), liver, and tibialis, were collected from the dams, and the placenta and liver were taken from the fetuses.

In the second experiment, an amino acid and lipid uptake study was performed in the same animal model of HF/HS diet (wt *n* = 6 dams and 38 placentas/fetuses, APNtg *n* = 4 dams and 28 placentas/fetuses). Tracers of L‐[1,2‐14C]‐Alanine (2 μCi/mouse; Perkin Elmer, Boston, MA, USA) and [9,10‐3H(N)]‐triolein (2 μCi/mouse, Perkin Elmer, Boston, MA, USA) were mixed in intralipid emulsion. The same procedure was carried out as mentioned above. After 30 min, the mice were killed, and tissues from the dam and fetuses were collected.

All the tissues were weighed (∼50–100 mg) and homogenized in 1 mL of a 2:1 chloroform: methanol solution using TissueLyzer (Qiagen) (this procedure is the same for both experiments). Samples were kept at 4°C overnight before 0.5 mL of 1 M CaCl_2_ was added and centrifuged at 3000 rpm for 20 min. The aqueous phase (oxidized fraction) was transferred to the scintillation vials containing 5 mL of Ultima GoldTM scintillation fluid (PerkinElmer, Waltham, Massachusetts, USA) while the organic phase (containing neutral lipids) was vaporized before scintillation fluid was added. Incorporation of ^3^H‐triolein, ^14^C‐glucose, and ^14^C‐alanine was quantified in a beta‐counter (Perkin Elmer, USA), and the results are expressed as a fraction (%) of total ^3^H or ^14^C counts per minute per gram of tissue (%CPM/g).

### 2.5 Serum Measurements

Fasted blood glucose was measured using a glucose analyzer (Contour Next, Bayer Health Care, Mishawaka, USA). Fasted blood samples were taken from dams at GD 18.5 to measure serum insulin (10‐1247‐01, Mercodia, Sweden) and total and high molecular weight (HMW) adiponectin using ELISA (47‐ADPMS‐E01, Alpco, USA). Serum adiponectin was measured in non‐fasted newborn mice within 2 h of birth in a separate cohort (80 659, Crystal Chem, USA). Serum triglycerides were analyzed using the TR210 kit (Randox, Crumlin, UK) with the following changes to the manufacturer's instructions: 200 μL of reagent R1 was added to each well containing 2 μL standard or serum in duplicates. A plate reader (SoftMax pro 7.0) was used to measure the absorbance. Serum concentrations of androstenedione, progesterone, 17‐alpha‐hydroxyprogesterone, testosterone, dihydrotestosterone, 17β‐estradiol, and estrone were determined with a validated liquid chromatography/mass spectroscopy (LC–MS/MS) [32].

### 2.6 Liver and Placenta Triglyceride Content

Liver triglycerides were extracted from the dams and fetuses; 30–40 mg of the liver was washed in 0.5 mL ice‐cold PBS and then homogenized for 2 min in 5% IGEPAL (CA‐630, Sigma Aldrich) diluted in distilled water using a TissueLyser (Qiagen, Hilden, Germany). The samples were then heated to 80°C for 5 min, then cooled to room temperature. This was repeated once before samples were centrifuged for 2 min at 14 800× *g*. The supernatant was transferred to a new tube and analyzed using the triglyceride kit TR210 (Randox, Cumlin, UK) as described above. Placenta triglycerides were extracted from 20 to 30 mg tissue homogenized for 5 min in 800 μL of a 2:1 chloroform: methanol (v/v) mixture using a TissueLyser, and then left on a nutating mixer for 20 min at room temperature. The samples were centrifuged at 16 200× *g* for 2 min and then 160 μL 0.9% NaCl was added before being centrifuged at 400× *g* for 2 min. 200 μL of the lower phase was collected and left to evaporate. The dried samples were resuspended in 75 μL isopropanol (Sigma‐Aldrich, Merck life science AB, Solna, Sweden) and analyzed using the Randox triglyceride kit (TR210).

### 2.7 Proteomic Analysis

Pools of two placentas or two livers from one male and one female fetus with the same genotype (wt or APNtg) from the same dam on HF/HS diet were used in proteomic analysis. A total of five pools per group (wt‐wt, wt‐APNtg, APNtg‐wt, and APNtg‐APNtg) were included in the liquid chromatography–tandem mass spectrometry (LC–MS/MS) analysis.

Sample preparation: Samples were homogenized using lysis matrix D (1.4 mm ceramic spheres) on a FastPrep‐24 instrument (MP Biomedicals, OH, USA) for 5 repeated 40‐s cycles at 6.5 m/s in a buffer containing 2% sodium dodecyl sulfate, 50 mM triethylammonium bicarbonate, and Pierce Phosphatase inhibitor (A32957, Thermo Fischer Scientific, Waltham, MA, USA). Lysed samples were centrifuged at 21 100× *g* for 10 min, and the supernatants were transferred to clean tubes. Protein concentrations in the lysates were determined using Pierce BCA Protein Assay Kit (Thermo Fischer Scientific) and the Benchmark Plus microplate reader (Bio‐Rad Laboratories, Hercules, CA, USA) with bovine serum albumin solutions as standards.

Aliquots containing 250 μg of total protein from each sample were incubated at 37°C for 60 min in the lysis buffer with DL‐dithiothreitol at 100 mM final concentration. The reduced samples were processed using the modified filter‐aided sample preparation method [33]. In short, the reduced samples were diluted to 1:4 by 8 M urea solution, transferred onto Nanosep 30 k Omega filters (Pall Corporation, Port Washington, NY, USA), and washed 4 times by adding 200 μL of 8 M urea and subsequent centrifugation at 13 800× *g*. Free cysteine residues were modified using 10 mM methyl methanethiosulfonate solution in digestion buffer (0.5% sodium deoxycholate, 50 mM triethylammonium bicarbonate) for 30 min at room temperature and the filters were then repeatedly washed with 100 μL of digestion buffer. Pierce trypsin protease (MS Grade, Thermo Fisher Scientific) in digestion buffer was added at a ratio of 1:100 relative to total protein mass and the samples were incubated at 37°C for 3 h; another portion of trypsin (1:100) was added and the mixture was incubated at 37°C overnight. The peptides were collected by centrifugation and labeled using Tandem Mass Tag (TMT 11plex) reagents (Thermo Fischer Scientific) according to the manufacturer's instructions. The labeled samples were combined into one pooled sample and concentrated using vacuum centrifugation, and sodium deoxycholate was removed by acidification with 10% trifluoroacetic acid and subsequent centrifugation. The labeled pooled sample was treated with Pierce peptide desalting spin columns (Thermo Fischer Scientific) according to the manufacturer's instructions.

Out of 2.8 mg of peptide material in the pooled labeled sample of each set, an aliquot corresponding to 300 μg was withdrawn for the total proteome analysis, an aliquot of 1.2 mg was subjected to phosphopeptide enrichment using the High‐Select Fe‐NTA Enrichment Kit, and another 1.2 mg aliquot was treated with the High‐Select TiO2 Phosphopeptide Enrichment Kit (both Thermo Fisher Scientific) according to the manufacturer's instruction. The eluted phosphopeptide samples were pooled.

For the total proteome analysis, the corresponding aliquot was fractionated into 40 primary fractions by basic reversed‐phase chromatography using a Dionex Ultimate 3000 UPLC system (Thermo Fischer Scientific). Peptide separations were performed on a reversed‐phase XBridge BEH C18 column (3.5 μm, 3.0 × 150 mm, Waters Corporation) using a linear gradient from 3% to 40% solvent B over 18 min followed by an increase to 100% B over 5 min and hold at 100% B for 5 min. Solvent A was 10 mM ammonium formate buffer at pH 10.00 and solvent B was 90% acetonitrile, 10% 10 mM ammonium formate at pH 10.00. The primary fractions were concatenated into final 20 fractions (1 + 21, 2 + 22, … 20 + 40), evaporated and reconstituted in 15 μL of 3% acetonitrile, 0.2% formic acid for nanoflow LC–MS analysis. The enriched phosphopeptide sample was fractionated into 20 primary fractions on the same LC setup with the gradient from 3% to 40% solvent B over 18 min, from 40% to 100% B over 5 min and 100% B for 7 min, the primary fractions were concatenated into final 20 fractions (1 + 11, 2 + 12, … 10 + 20), evaporated and reconstituted in 15 μL of 3% acetonitrile, 0.2% formic acid for nanoflow LC–MS analysis.

LC–MS/MS Analysis: The fractions were analyzed on an Orbitrap Fusion Tribrid mass spectrometer interfaced with an Easy‐nLC 1200 liquid chromatography system (Thermo Fisher Scientific). Peptides were trapped on an Acclaim Pepmap 100 C18 trap column (100 μm × 2 cm, particle size 5 μm, Thermo Fischer Scientific) and separated on an analytical column (75 μm × 35 cm, packed in‐house with Reprosil‐Pur C18, particle size 3 μm, Dr. Maisch, Ammerbuch, Germany) at a flow of 300 nL/min using 0.2% formic acid in water as solvent A and 80% acetonitrile, 0.2% formic acid as solvent B.

For the total proteome analysis, peptides were eluted using a linear gradient from 5% to 35% B over 75 min followed by an increase to 100% B over 5 min and a hold at 100% B for 10 min. MS scans were performed at 120 000 resolution in the m/z range 380–1380. The most abundant doubly or multiply charged precursors from the MS1 scans were isolated using the quadrupole with 0.7 m/z isolation window with a “top speed” duty cycle of 3 s and dynamic exclusion within 10 ppm for 60 s. The isolated precursors were fragmented by collision‐induced dissociation at 35% collision energy with the maximum injection time of 50 ms and detected in the ion trap, followed by multinotch (simultaneous) isolation of the top 5 MS2 fragment ions within the m/z range 400–1400, fragmentation (MS3) by higher‐energy collision dissociation (HCD) at 65% collision energy and detection in the Orbitrap at 50 000 resolution, m/z range 100–500 and maximum injection time 105 ms.

For the phosphorylation analysis, peptides were eluted using a linear gradient from 5% to 35% B over 75 min followed by an increase to 100% B over 5 min and a hold at 100% B for 10 min. MS scans were performed at 120 000 resolution in the m/z range 380–1380. The most abundant doubly or multiply charged precursors from the MS1 scans were isolated using the quadrupole with 0.7 m/z isolation window with a “top speed” duty cycle of 3 s and dynamic exclusion within 10 ppm for 60 s. The isolated precursors were fragmented by higher‐energy collision dissociation (HCD) at 38% collision energy and the MS2 spectra were detected in the Orbitrap at 50 000 resolution, with the fixed first m/z 100 and maximum injection time 105 ms.

Proteomic data analysis: Identification and relative quantification were performed using Proteome Discoverer version 2.4 (Thermo Fisher Scientific). The database search was performed using the Mascot search engine v. 2.5.1 (Matrix Science, London, UK) against the Swiss‐Prot *Mus musculus* database. Trypsin was used as a cleavage rule with no or one missed cleavage allowed for the total or phosphoproteome, respectively. Methylthiolation on cysteine residues and TMT at peptide N‐termini and on lysine side chains were set as static modifications, and oxidation on methionine was set as a dynamic modification. For the total proteome analysis, precursor mass tolerance was set at 5 ppm and fragment ion tolerance at 0.6 Da. For the phosphopeptide analysis, precursor mass tolerance was set at 5 ppm and fragment ion tolerance at 0.02 Da; phosphorylation on serine, threonine, and tyrosine was set as an additional dynamic modification. Percolator was used for PSM validation with the strict FDR threshold of 1% in both cases. Quantification was performed in Proteome Discoverer 2.4. TMT reporter ions were identified with 3 mmu mass tolerance in the MS3 HCD spectra for the total proteome experiment and with 20 ppm mass tolerance in the MS2 HCD spectra for the phosphopeptide experiment. Only the unique identified peptides were considered for the protein quantification. The mass spectrometry proteomics data have been deposited to the ProteomeXchange Consortium via the PRIDE [34] partner repository with the dataset identifier PXD025390.

Data processing: Total and phosphoprotein TMT abundance values were analyzed in R version 4.0.2 [35]. Data were first log2 transformed and filtered to remove proteins without gene symbols. For phosphoprotein data, peptides were mapped to genes using the *org.Mm.eg.db* package in R [36]. Next, proteins with missing values for all samples and duplicate proteins were removed. For duplicates, only the protein with the highest mean abundance was retained. Abundance values were then adjusted with ComBat [37] to correct for technical variation between the two 10‐plex TMT runs. Quality control of corrected data was performed by inspecting the distribution, sample correlations, and principal component analysis. For the phosphoprotein data, one sample (L33) was identified as an outlier and removed. Differential protein expression analysis was performed with *limma* [38], and *p* values were adjusted for multiple testing using Benjamini–Hochberg correction. Proteins where FDR < 5% and log2 fold change > 0.15 were considered significant. Gene ontology enrichment analysis of differentially expressed proteins was performed with STRING to identify significantly enriched signaling pathways (https://string‐db.org/). Ontology terms with a *q*‐value < 0.05, strength score > 0.25, and including at least 3 proteins were considered as enriched. Processing and functional analysis of phosphoproteomic data was analyzed using PhosR to identify kinase–kinase interactions (kinase hubs were defined as kinases that interacted with three or more other kinases) and kinase–substrate pairs to find the kinases most likely to regulate a phosphosite [39]. The PhosR analysis is published at https://github.com/Saharsoli1393/Phosphoprotein‐analysis‐by‐PhosR‐package‐in‐R.

### 2.8 Statistical Analyses

Data are presented as the mean ± SEM and were analyzed with RStudio (R version 4.0.2) and Prism (version 9.2.0). The main effect of diet‐induced obesity and adiponectin overexpression on fetal group (wt‐wt, wt‐APNtg, APNtg‐wt, APNtg‐APNtg) or dam genotype (wt, APNtg) were measured using two‐way ANOVA followed by Tukey post hoc test. Tracer uptake data was not normally distributed and was therefore analyzed by Mann–Whitney *U* test. Correlations between circulating adiponectin, bone mineral content and fetal weight were analyzed using Spearman correlation. Data are presented as mean ± sem and *p* < 0.05 was considered significant.

## 3 Results

### 3.1 Dam Characteristics

Female mice from wt and APNtg were fed a normal diet (wt *n* = 7, APNtg *n* = 6) or a HF/HS diet (wt *n* = 9, APNtg *n* = 8) for 8 weeks prior to mating (Figure 1). Female mice fed the HF/HS diet were heavier and had increased fat mass and lean mass compared to mice on the normal diet prior to mating (Table S1). Bone mineral content was lower in APNtg dams (Table S1), and serum adiponectin was inversely correlated with bone mineral content (r = −0.422, *p* = 0.025), aligning with previous research [40].

On average, two mating attempts were made to obtain pregnant dams, and there was no difference between genotypes or diets. Pregnant wt mice on an HF/HS diet had similar body and tissue weights as wt mice on NC but displayed an increased liver weight (Table 1). APNtg dams on the HF/HS diet had a higher body weight, liver weight, and more inguinal fat compared to APNtg dams on NC. The increased fat pad weight is consistent with the effect of adiponectin to store excess calories in adipose tissue to maintain metabolic flexibility [7]. There was a main effect of adiponectin on fasting blood glucose and insulin, with lower levels in APNtg mice before mating (Table S1), but there was no significant difference in blood glucose in pregnant dams at GD 18.5 (Table 1). We identified the characteristic increase in brown adipose tissue in APNtg mice compared to wt mice [41], and this effect was even more pronounced in APNtg dams on the HF/HS diet, while there was no effect of the HF/HS diet on brown adipose tissue weight in wt dams (Table 1).

**TABLE 1:** Litter size, body weight, tissue weights, and serum measurements at GD18.5 in adiponectin transgenic (APNtg) and wild‐type (wt) dams on high fat/high sucrose (HF/HS) diet or normal chow (NC).

	wt NC (*n* = 7)	APNtg NC (*n* = 6)	wt HF/HS (*n* = 9)	APNtg HF/HS (*n* = 8)	Two‐way ANOVA
Litter size (*n*)	8.00 ± 0.49	7.17 ± 0.91	6.11 ± 0.87	6.88 ± 0.83	ns
Absorbed fetuses at implantation sites (*n*)	0.43 ± 0.2	0.67 ± 0.5	0.78 ± 0.2	1.25 ± 0.5	ns
Body weight (g)	35.69 ± 0.97	33.18 ± 1.03	34.38 ± 0.84	41.88 ± 3.00*	*F*_(1,26)_ = 4.15^a^ ns, *p* = 0.052 (i)
Heart (mg)	115.1 ± 3.4	110.9 ± 5.5	113.9 ± 1.9	139.4 ± 3.8*	*F*_(1,26)_ = 5.86^a^ *p* = 0.023 *F*_(1,26)_ = 9.99^b^ *p* = 0.004 (i)
Ovaries (mg)	15.4 ± 1.5	14.5 ± 1.4	14.1 ± 1.6	15.5 ± 1.2	ns
Inguinal fat (mg)	452.9 ± 40.0	242.6 ± 20.9	616.6 ± 47.9	817.5 ± 229.5*	*F*_(1,26)_ = 58.14^a^ *p* = 0.008
Mesenteric fat (mg)	194.5 ± 14.8	145.8 ± 21.4	233.5 ± 36.0	215.7 ± 48.1	ns
BAT (mg)	65.1 ± 5.9	119.1 ± 15.6	58.6 ± 2.8	293.0 ± 59.9*	*F*_(1,26)_ = 6.34^a^ *p* = 0.018 *F*_(1,26)_ = 18.83^b^ *p* = 0.0002 (i)
Pancreas (mg)	122.9 ± 4.7	118.0 ± 7.7	115.8 ± 7.5	112.9 ± 7.6	ns
Liver (g)	1.48 ± 0.09	1.28 ± 0.05	1.98 ± 0.05*	2.36 ± 0.13*	*F*_(1,26)_ = 78.61^a^ *p* < 0.0001 (i)
Kidney (mg)	244.7 ± 6.3	234.0 ± 6.2	243.6 ± 5.5	261.2 ± 11.8	ns
Adrenals (mg)	4.7 ± 0.3	5.5 ± 0.5	5.8 ± 0.4	7.2 ± 0.8	*F*_(1,26)_ = 6.08^a^ *p* = 0.021
b‐glucose (mmol/L)	6.1 ± 0.4	5.5 ± 0.3	5.8 ± 0.6	5.3 ± 0.2	ns

*Note:* The effect of diet‐induced obesity and adiponectin overexpression on dam group (wt and APNtg) was measured using two‐way ANOVA followed by Tukey post hoc test. *p* < 0.05 vs. wt within diets, *p < 0.05 vs. HF/HS diet within genotypes. Data are presented as mean ± SEM.

Abbreviations: BAT, brown adipose tissue; i, interaction effect; ns, not significant.

^a^ Main effect of diet.

^b^ Main effect of genotype.

### 3.2 Serum, Liver, and Placenta Triglycerides

**FIGURE 2:**
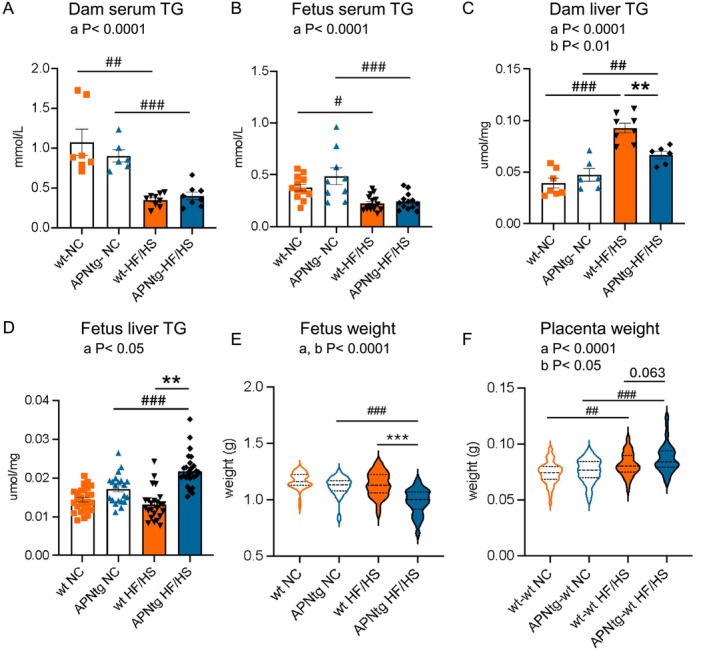
(A) Serum triglyceride (TG) levels in adiponectin transgenic (APNtg) and wild‐type (wt) dams and (B) in fetuses on normal chow (NC) and high fat/high sucrose (HF/HS) diet. (C) Liver triglyceride content in dams and (D) in fetuses on NC or HF/HS diet. (E) Fetal weight, and (F) placental weight, at gestational day (GD) 18.5 in APNtg and wt dams on normal chow (NC) or high fat/high sucrose (HF/HS) diet. The effect of diet‐induced obesity and adiponectin overexpression was analyzed using two‐way ANOVA followed by Tukey post hoc test, a; main effect of diet, and b; main effect of dam genotype (wt vs. APNtg). **p < 0.01, ***p < 0.001 for the effect of dam genotype within diet groups, ^#^ *p* < 0.015, ^##^ *p* < 0.01, ^###^ *p* < 0.001 for the effect of diet within dam genotype groups as analyzed by Tukey post hoc test. There was a significant interaction effect for fetus liver weight (*F*_(3,91)_ = 7.098, *p* < 0.001) and fetus weight (*F*_(3,199)_ = 6.583, *p* < 0.001). Data are presented as mean ± SEM and includes 54 fetuses from 7 wt dams and 42 fetuses from 6 APNtg on NC, and 46 fetuses from 9 wt dams and 55 fetuses from 8 APNtg on a HF/HS diet.

Serum triglycerides were lower in APNtg and wt dams on HF/HS diet compared with NC (*F*_(1,26)_ = 47.9, *p* < 0.0001) (Figure 2A), with no difference between genotypes. A similar pattern was seen in fetuses, where fetuses from dams on HF/HS diet had lower serum triglycerides compared to fetuses from dams on NC (*F*_(1,46)_ = 27.6, *p* < 0.0001) (Figure 2B). The opposite was detected in the liver, where liver triglyceride content was higher in APNtg and wt dams on HF/HS diet compared with NC (*F*_(1,24)_ = 56.9, *p* < 0.0001). However, the APNtg dams on HF/HS diet had lower liver triglycerides compared with wt HF/HS, likely due to the increased capacity for fat storage in adipose tissue (Figure 2C). In contrast, fetuses from APNtg dams had more liver triglycerides compared to fetuses from wt HF/HS fed dams (Figure 2D, Figure S12). This suggests that wt dams on the HF/HS diet were better at protecting their fetuses from fatty acid overload. Placenta triglyceride content was similar between genotypes (wt 3.2 ± 0.5 vs. APNtg 3.5 ± 0.6 nmol/mg placenta, *F*_(1,37)_ = 0.2, *p* = 0.65) and was not altered by the HF/HS diet (wt 3.2 ± 0.3 vs. APNtg 3.5 ± 0.2 nmol/mg placenta, *F*_(1,37)_ = 0.03, *p* = 0.86).

### 3.3 Fetus Characteristics

Litter size was comparable between groups, and the number of implantation sites with absorbed fetuses was similar (Table 1). There was a main effect of diet and dam genotype on fetal weight (Diet *F*_(1,203)_ = 31.9, *p* < 0.0001, Fetal group *F*_(1,203)_ = 46.1, *p* < 0.0001) (Figure 2E). APNtg dams on an HF/HS diet carried fetuses with a lower weight compared to fetuses from APNtg dams on NC and compared to wt dams on the HF/HS diet (Figure 2E). The HF/HS diet in wt dams did not alter fetal weight (Figure 2E). This is in contrast to a previous study showing that this diet leads to fetal overgrowth [42], a phenotype that we are unable to replicate [17, 43, 44]. Maternal overexpression of adiponectin did not alter fetal weight in dams on NC (Figure 2E), and fetal overexpression of adiponectin had no effect on fetal weight compared with wt littermates (Figure S1A). There was a small main effect of dam genotype on placenta weight (*F*_(1,197)_ = 5.2, *p* < 0.05), while the HF/HS diet increased placenta weight in both APNtg and wt dams (*F*_(1,197)_ = 35.2, *p* < 0.0001) (Figure 2F). Previous studies have shown that higher placenta weight is a predictor of maternal hypertension and diabetes mellitus and is associated with a poor perinatal outcome [45]. Fetal overexpression of adiponectin had no effect on placenta weight or fetal/placenta weight ratio compared to wt littermates (Figure S1B,C). Male fetuses had larger placentas than female fetuses; otherwise, there was no difference between sexes (Table S2).

### 3.4 Serum Adiponectin and Sex Hormones

**FIGURE 3:**
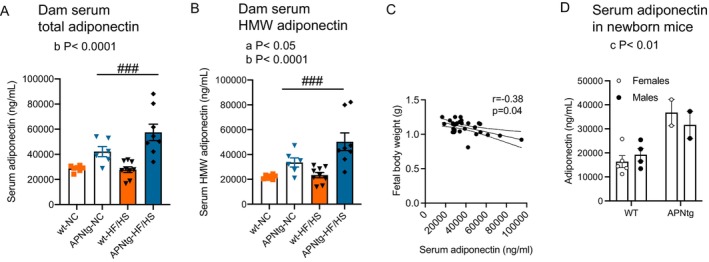
(A) Serum total and (B) high molecular weight (HMW) adiponectin levels in adiponectin transgenic (APNtg) and wild‐type (wt) dams on normal chow (NC) or high fat/high sucrose (HF/HS) diet (*n* = 7 wt, *n* = 6 APNtg on NC, *n* = 9 wt, *n* = 8 APNtg on HF/HS diet). (C) Spearman correlation between dam adiponectin levels and fetal weight. (D) Serum total adiponectin in newborn mice within 2 h from birth (*n* = 5 wt females, *n* = 4 wt males, *n* = 2 APNtg females, *n* = 2 APNtg males). The effect of diet‐induced obesity and adiponectin overexpression was measured using two‐way ANOVA followed by Tukey post hoc test, a; main effect of diet, and b; main effect of dam group (wt vs. APNtg). *p < 0.05 for the effect of dam genotype within diet groups, ^###^ *p* < 0.001 for the effect of diet within dam genotype groups as analyzed by Tukey post hoc test. The effect of **sex** and adiponectin overexpression on serum adiponectin was measured using two‐way ANOVA, c; main effect of APNtg genotype. There was no interaction effect. Data are presented as mean ± SEM.

The HF/HS diet did not alter circulating total and high molecular weight (HMW) adiponectin in wt dams (Figure 3A,B). As expected, APNtg dams had 40%–50% higher circulating HMW and total adiponectin compared to wt dams, and HF/HS diet further increased adiponectin in APNtg dams (Figure 3A,B). HMW adiponectin, suggested to mediate most of the biological effects, was the most abundant form. Total adiponectin levels showed a negative correlation with fetal weight (Figure 3C), indicating an inhibitory effect of adiponectin on fetal growth. Serum adiponectin is higher in females than in males, but this difference plays a minor role during fetal development, since in mice, adiponectin expression begins during late fetal development, coinciding with the formation and maturation of adipocytes [18]. To determine if there was any difference between males and females at birth, we measured serum adiponectin in newborn mice (0‐2 h postnatally). Newborn APNtg mice had higher serum adiponectin compared with wt littermates, but there was no difference in serum adiponectin between males and females (Figure 3D, *p* = 0.77 for the effect of sex). Serum concentrations of sex hormones were analyzed in dams at GD 18.5. There was no difference in circulating estradiol or testosterone between wt and APNtg dams (Table 2), indicating that the difference in fetal growth between wt and APNtg dams is not driven by these sex hormones. On the other hand, there was a main effect of diet on higher progesterone and lower testosterone in HF/HS‐fed dams (Table 2). There was a negative correlation between fetal weight and progesterone levels (*r* = −0.576, *p* = 0.001), supporting the idea that progesterone has a negative impact on glucose uptake in the placenta [46].

**TABLE 2:** Sex hormone serum measurements at GD18.5 in adiponectin transgenic (APNtg) and wild‐type (wt) dams on high fat/high sucrose (HF/HS) diet or normal chow (NC).

Sex hormones (pg/mL)	wt‐NC (*n* = 7)	APNtg‐NC (*n* = 6)	Wt‐HF/HS (*n* = 9)	APNtg‐HF/HS (*n* = 8)	Two‐way ANOVA
Androstenedione	405 ± 55	334 ± 56	294 ± 46	412 ± 36	ns
17‐alpha‐hydroxyprogesterone	87 ± 14	69 ± 14	97 ± 16	121 ± 8	*F*_(1,26)_ = 5.09^a^ *p* = 0.033
Testosterone	329 ± 65	258 ± 44	122 ± 16	183 ± 17	*F*_(1,26)_ = 13.86^a^ *p* = 0.001
Dihydrotestosterone	33 ± 8	17 ± 1	< 13	19 ± 2	ns
Estradiol	36 ± 4	38 ± 8	30 ± 6	23 ± 3	ns
Estrone	12 ± 2	12 ± 3	8 ± 2	9 ± 2	ns
Progesterone	4191 ± 1075	8650 ± 6991	22 950 ± 9781	48 579 ± 12 916	*F*_(1,26)_ = 9.24^a^ *p* = 0.005

*Note:* The effect of diet‐induced obesity and adiponectin overexpression on dam group (wt and APNtg) was measured using two‐way ANOVA followed by Tukey post hoc test. Data are presented as mean ± SEM.

^a^ Main effect of diet, there was no interaction effect.

### 3.5 Glucose, Lipid, and Amino Acid Tracer Uptake in Dams and Fetuses

**FIGURE 4:**
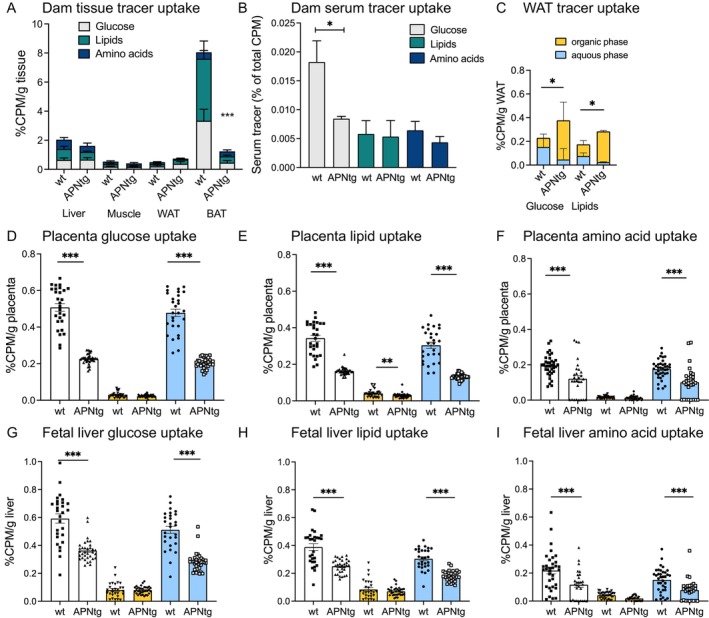
Glucose, lipid, and amino acid tracer uptake in (A) tissues and (B) serum from adiponectin transgenic (APNtg) and wild‐type (wt) pregnant dams on HF/HS diet at Gestational Day 17.5. (C) Glucose and lipid tracer uptake in white adipose tissue (WAT) from dams (wt *n* = 4, APNtg *n* = 5). (D) Glucose, (E) lipid, and (F) amino acid tracer uptake in placenta. (G) Glucose, (H) lipid, and (I) amino acid tracer uptake in fetal liver (wt *n* = 28–38, APNtg *n* = 28–32). White bars represent total tracer uptake, yellow bars are tracer in the organic phase and blue bars the aqueous phase. *p < 0.05, **p < 0.01, ***p < 0.001 for the effect of maternal adiponectin overexpression as analyzed by Mann–Whitney U test. Data are presented as mean ± SEM.

Since the effect on fetal growth was most pronounced in dams on an HF/HS diet, we performed tracer studies to analyze tissue‐specific nutrient uptake in this setting. The first cohort included glucose and lipid tracers, and the second cohort included amino acid and lipid tracers. Glucose and lipid uptake in liver, skeletal muscle, and white adipose tissue was similar between dam genotypes, while the brown adipose tissue uptake was lower in APNtg dams (Figure 4A). Although the total glucose and lipid uptake in adipose tissue was similar between genotypes, APNtg dams had increased clearance of glucose from the circulation (Figure 4B) and increased white adipose tissue incorporation of ^14^C‐glucose and ^3^H‐triolein tracer in the organic phase (Figure 4C). This, in combination with the reduced hepatic triglyceride accumulation (Figure 2C) and increased subcutaneous fat gain during gestation (Table 1), indicates that APNtg dams have an enhanced ability to store excess nutrients as subcutaneous fat compared with wild types.

The placental uptake of glucose, lipid, and amino acid tracer was significantly reduced in APNtg compared to wt dams (Figure 4D–F). Glucose tracer was mainly in the aqueous phase, with < 10% of glucose metabolites in the organic phase, indicating that glucose metabolites are not stored as neutral lipids in the placenta (Figure 4D). Lipid tracer was mainly found in the aqueous phase, representing metabolized fatty acids, while the organic phase with the intact fatty acids (oleic acids) of the tracer accounted for 16% and 27% of the total tracer uptake in wt and APNtg placentas, respectively (Figure 4E). The water‐soluble amino acid tracer was predominantly found in the aqueous phase (Figure 4F) indicating that amino acids are utilized for energy but not used for de novo lipogenesis and stored as fats in the placenta. Interestingly, APNtg dams showed a lower amount of amino acid tracer in the aqueous phase of both placenta and fetal liver, indicating a lower uptake in the placenta and hence less available amino acid for the fetus. Since the tracer concentration in the placenta is similar to those in the fetal livers, negligible amounts of tracers are extensively metabolized or stored in the placenta (Figure 4F,I). The uptake of glucose and lipid tracer was also significantly reduced in the liver of fetuses from APNtg dams (Figure 4G,H). The difference was due to decreased amounts of glucose and lipid tracers in the aqueous phase in fetuses from APNtg dams, since there were equal amounts of tracer in the organic phase (Figure 4G,H). Therefore, a larger proportion of the total amount of glucose and lipid uptake in fetuses from APNtg dams is stored as fat in the liver.

### 3.6 Total and Phosphorylated Protein Expression in Placenta

**FIGURE 5:**
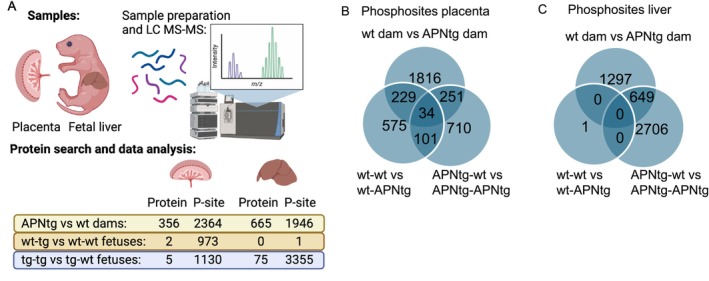
(A) Overview of proteomics analysis and data. The number of significantly expressed proteins and differentially phosphorylated sites (P‐sites) in placenta and fetal liver analyzed between dam genotypes (APNtg vs. wt, *n* = 10/group) and within fetal genotypes (wt‐tg vs. wt‐wt and tg‐tg vs. tg‐wt, *n* = 5/group). Overlap of differently phosphorylated sites in (B) placenta and (C) fetal liver (dam genotypes and fetal genotypes). Differently expressed proteins and phosphorylated sites have a log2 fold change > 0.15 or < −0.15, and *q* < 0.05.

Total protein expression in placenta was analyzed from APNtg and wt dams on HF/HS diet to determine which pathways were altered in response to elevated adiponectin. A total of 7290 proteins were identified, and after correction for multiple testing, 356 unique proteins with FDR < 0.05 were found to be differentially expressed in the placenta of APNtg dams versus wt (Figure 5A, Table S3A). In total, 136 proteins were upregulated and 220 downregulated in the placenta of APNtg dams, and the log fold change ranged from 2.76 to −4.19. As expected, adiponectin was one of the most upregulated proteins, together with another adipokine, adipsin (complement factor D), a complement‐activating factor that may play a role in spontaneous abortion [47]. Lipoprotein lipase was more abundantly expressed in placentas from APNtg dams while several amino acid transporters had a lower expression: SLC1A4, SLC3A2, and SLC7A1 (Table S3A). The vitamin B transporter SLC5A6 was also downregulated. Animal studies have shown that a deficiency of vitamin B7 (biotin) during pregnancy leads to embryonic growth retardation [48]. Other proteins involved in placenta function include hemopexin and fibronectin leucine‐rich transmembrane protein 2, which regulate early embryonic vascular and neural development [49]. Finally, we investigated if protein expression was different between APNtg and wt fetuses from wt and APNtg dams, respectively. In wt dams, APNtg fetuses had two differentially expressed proteins, and in APNtg dams, APNtg fetuses had five differentially expressed proteins (Table S3B,C). The low number of differentially expressed proteins in placentas from littermate wt and APNtg fetuses indicates that the maternal genotype is the main driver of placental protein expression.

**FIGURE 6:**
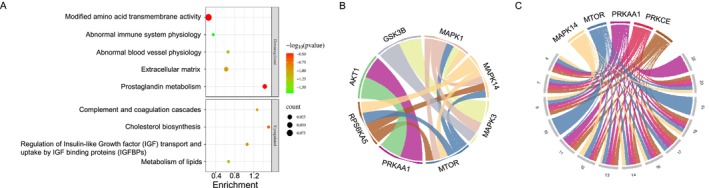
(A) Selected gene ontology enriched pathways (*q* < 0.05) of differentially expressed proteins in placentas from APNtg dams compared with wild type (*n* = 10/group). (B) Predicted kinase–kinase interaction network and (C) signalome in placentas.

We tested whether groups of biologically related proteins were altered in the APNtg compared to wt placentas and found significant enrichment in 37 upregulated and 28 downregulated pathways (Table S4). Upregulated proteins were involved in lipid metabolism, cholesterol biosynthesis, complement and coagulation cascade, and insulin‐like growth factor‐1 (IGF‐1) signaling (Figure 6A, Table S4). The higher expression of proteins enriched in the “Regulation of IGF transport and uptake by IGF binding proteins (IGFBPs)” pathway in the placentas from APNtg dams aligns with the idea is that adiponectin suppresses fetal growth by increasing the IGFBP‐1 in placental trophoblasts, leading to a decrease in IGF‐1 availability for the fetus (20, 22). Downregulated proteins were involved in amino acid transport, immune system processes, and the physiology of abnormal blood vessels (Figure 6A, Table S4).

Next, we analyzed phosphorylation sites (p‐sites) in placenta proteins. In total, 19 264 sites were identified and 2364 p‐sites in 1548 unique proteins remained significant after correction for FDR < 0.05 (Table S5A). Less than 20% (17%) of the differentially expressed proteins had at least one altered phosphorylation site. 34 p‐sites were consistently altered when overlapping APNtg vs. wt dams and APNtg vs. wt fetuses (Figure 5B). Two thirds of these sites showed a higher phosphorylation in placentas from APNtg dams vs. wt and were higher in APNtg fetuses vs. wt fetuses from these dams (Table S5B,C). One of these sites was phosphorylation of serine 2448 in mTOR, which was increased in placentas from APNtg dams (0.26 log fold change vs. wt, Table S5A). This site had even higher phosphorylation in placentas from APNtg fetuses vs. wt fetuses from APNtg dams (0.25 log fold change, Table S5C). This mTOR phosphorylation site is the most well‐described and is associated with mTOR signaling [50]. We identified three so‐called kinase hubs, predicted kinases that interact with several kinases in the placenta (Figure 6B). Thus, MAPK1, MAPK14, and MTOR play a central roles in phosphorylation processes critical for placental function. PRKAA1 and AKT1 are exclusively connected, suggesting a specific regulatory interaction between these two kinases. In response to nutrients, growth factors, or amino acids, mTOR phosphorylates EIF4EBP1 (Thr69) and phosphorylates and activates RPS6KB1 (Ser427) and RPS6KB2 (Ser423) via mTORC1, sites that showed increased phosphorylation in placentas from APNtg dams (Table S5A). GSK3B displays specific connectivity to MAPK3 (Figure 6B). GSK3B acts as a negative regulator in the hormonal control of glucose homeostasis by phosphorylating and inactivating glycogen synthase 1 (GYS1), thereby inhibiting glycogen synthesis. It similarly suppresses protein synthesis by controlling the activity of initiation factor 2B (EIF2B) (UniProt.org). GYS1 exhibited increased phosphorylation at Thr714, Thr722, Thr724, Ser728, and Thr730 while EIF2B had increased phosphorylation at Ser103 in placentas from APNtg dams (Table S5A). The placenta signalome shows that maternal overexpression of adiponectin is associated with 14 protein modules (*p* < 0.05, Table S6A), each of which has a specific phosphorylation. These modules are regulated by five key kinases: MAPK14, MTOR, PRKAA1, PRKCE, and RPS6KA5 (Figure 6C, Table S6B). A heatmap of the kinase–substrate pairs for the predicted top p‐sites of the evaluated kinase in the placenta can be found in Figure S2.

### 3.7 Total and Phosphorylated Protein Expression in the Fetal Liver

**FIGURE 7:**
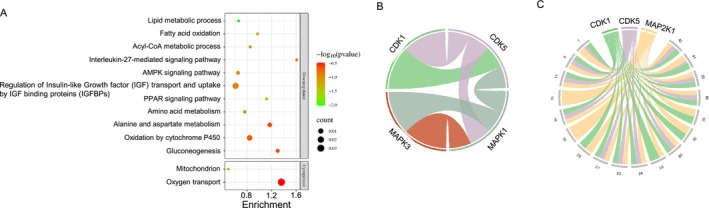
(A) Selected gene ontology enriched pathways (*q* < 0.05) of differentially expressed proteins in fetal livers from APNtg dams compared with wild type (*n* = 10/group). (B) Predicted kinase–kinase interaction network and (C) signalome in fetal livers.

Total protein expression in the fetal livers was analyzed from wt and APNtg fetuses from APNtg and wt dams fed HF/HS diet. A total of 7301 proteins were identified, and after correction for multiple testing, 665 unique proteins with FDR < 0.05 were found to be differentially expressed in fetal livers from APNtg dams vs. wt (Figure 5A). In total, 265 proteins were upregulated and 400 downregulated in fetal livers from APNtg dams, and the log fold change ranged from 1.86 to −2.16 (Table S7A). We tested whether sets of biologically related proteins were altered in fetal liver from APNtg mice compared with wt and found significant enrichment of 12 upregulated and 81 downregulated pathways (Table S8). For example, upregulated proteins were involved in mitochondrial translation, whereas downregulated proteins were enriched in fatty acid oxidation, amino acid metabolism, PPAR signaling, and IGF‐1 signaling pathways (Figure 7A, Table S8). Moreover, 75 proteins were differentially expressed in livers from APNtg fetuses compared with wt fetuses carried by APNtg dams (Figure 5A, Table S7B). Upregulated proteins were enriched in white fat cell differentiation (WP2872, strength 1.46, *q* = 0.048). CEBPB was one of these upregulated adipogenic transcription factors. In contrast, there was no difference in protein expression between APNtg and wt fetuses from wt dams.

In total, 15 334 p‐sites were identified in fetal liver proteins and of those, 1946 in 1389 unique proteins remained significantly different between genotypes after correction for FDR < 0.05 (Figure 5C, Table S9A). Only 6% of the differentially expressed proteins had at least one altered p‐site. Moreover, 3355 p‐sites were differentially expressed in livers from APNtg fetuses compared with wt fetuses carried by APNtg dams (Table S9B). Only one p‐site differed significantly between fetuses from wt dams; Ser219 in APOOL showed increased phosphorylation in APNtg fetuses (logFC 1.2, *q* = 0.03). This suggests that the effects of adiponectin overexpression on fetal liver get enhanced in APNtg fetuses from APNtg dams, while adiponectin‐overexpressing fetuses from wt dams have a similar proteome as their wt littermates. The kinase network visualization highlights the interconnected nature of 4 kinases with regulatory relationships in the fetal liver: CDK1, CDK5, MAPK3, and MAPK1 (Figure 7B). CDK1 is connected to CDK5 and MAPK3 is connected only to MAPK1, suggesting a focused interaction within the CDK and MAPK families. The signalome shows that maternal overexpression of adiponectin is associated with 18 protein modules (*p* < 0.05, Table S10A) each with a distinct phosphorylation. These modules are regulated by only three kinases: CDK1, CDK5, and MAP2K1 (Figure 7C, Table S10B). A heat map of the kinase–substrate pairs for the predicted top p‐sites of the evaluated kinases in fetal liver can be found in Figure S3A. From the kinase perturbation plot, we can conclude that AMPK shows a lower activity in fetal liver from wt compared with APNtg dams, in line with the finding that adiponectin increases insulin sensitivity through the AMPK activation in liver [22, 51] (Figure S3B).

The overlap in protein expression in liver and placenta showed that 30 proteins were differentially expressed in both tissues. Four proteins were upregulated and 16 downregulated in both placenta and fetal liver from APNtg dams (Table S11A). Comparison of gene ontology enrichment revealed lipid metabolism and IGF transport pathways upregulated in the placenta that were downregulated in the fetal liver (Table S11B).

## 4 Discussion

This study shows that elevated maternal adiponectin decreases fetal growth in obese but not normal weight dams by decreasing placental nutrient transport. Moreover, similar protein expression in placentas from wt and APNtg littermates indicates that maternal adiponectin levels are the main driver of placenta function and that maternal signals dominate over fetal demand signals. Fetal overexpression of adiponectin did not alter fetal growth. This may be due to the relatively late expression of adiponectin around Embryonic Day 15 or that adiponectin produced by the fetus mainly affects adipocyte development and liver metabolism [18]. Our findings regarding the effect of adiponectin on placenta function and fetal growth build on previous work by Jansson's and Powell's groups [19, 20, 22]. They treated normal weight and obese dams with recombinant adiponectin during the last 5 days of gestation to prevent fetal overgrowth and assess the effects on placenta function [19, 20]. Our approach allowed us to determine not only the effects of chronically elevated maternal adiponectin but also the contribution of fetal adiponectin.

Adiponectin cannot pass the placental barrier. Thus, maternal and fetal adiponectin are not interchangeable, and maternal adiponectin acts on the placenta and not directly on the fetus. Moreover, increased fetal adiponectin did not affect placenta protein expression, suggesting that the change in the placental metabolic function is driven by maternal adiponectin. However, fetal overexpression of adiponectin changed the phosphorylation of around 1000 proteins, indicating that maternal adiponectin is the dominant factor for protein expression in the placenta, while both maternal and fetal overexpression of adiponectin alter the phosphoproteome.

Elevated maternal adiponectin is associated with better metabolic health in adult offspring from normal weight and obese dams and can prevent fetal overgrowth when administrated during the rapid growth phase during the later part of gestation in both sexes [19, 21, 27]. This is in stark contrast to this study where chronically elevated adiponectin in obese dams resulted in reduced placenta nutrient transport, fetal growth restriction, and altered fetal liver function, effects that could lead to metabolic diseases later in life. Both sexes were equally susceptible to the negative impacts of elevated adiponectin. According to the DOHaD theory, decreased fetal nutrient supply not only results in children born with low birth weight or small for gestational age but also programs the fetus's metabolic health later in life [1, 2]. Thus, it is very important to consider the consequences of maternal obesity on nutrient transport and the time window of exposure when evaluating adiponectin as a treatment option.

### 4.1 Adiponectin and Placental Function

The increased glucose uptake in white adipose tissue in APNtg dams may have reduced the available glucose for the placenta, but our data suggest that reduced fetal growth is primarily due to reduced nutrient transport across the placenta. In humans, intrauterine growth restriction has been consistently associated with reduced amino acid transport across the placenta [52]. Moreover, downregulation of amino acid transporters precedes the development of growth restriction in mice, suggesting that this mechanism is a driving force and not a response to a decrease in fetal demand [52]. Our tracer study confirms that the uptake of alanine was 50% lower in placentas from adiponectin overexpressing dams. The placenta expresses over 25 different amino acid transporters, each responsible for the uptake of several different amino acids. We identified three amino acid transporters not previously shown to be downregulated by adiponectin. The newly identified neutral amino acid transporter A (SLC1A4) is a transporter for alanine, serine, cysteine, and threonine. The heavy chain amino acid transporter SLC3A2 forms a dimer with the well‐studied amino acid transporters LAT1 or LAT2 to take up leucine [20], and the high affinity cationic amino acid transporter 1 (CAT‐1/SLC7A1) transports arginine, lysine, and ornithine (uniprot.org), corroborating previous findings that adiponectin reduces the transport of amino acid transporters to the trophoblast plasma membrane, thereby reducing fetal amino acid availability [20].

Adiponectin has been shown to inhibit placental mechanistic target of rapamycin (mTOR), whereas maternal obesity activates it [22]. Not surprisingly, mTOR, a key player in placenta metabolism and cell growth [22, 52], was identified as one of the kinase hubs contributing to the altered phosphoproteome in placentas from adiponectin overexpressing dams. Inhibition of mTOR signaling is believed to mediate the reduced uptake of amino acids and may contribute to the growth restriction and reduced amino acid transport to the fetus [22, 52]. The phosphorylation of mTOR is regulated by several upstream stimuli, including adiponectin, growth factors (IGF‐1), and nutrients. Ser2448 on mTOR is a protein kinase B‐mediated phosphorylation site directly related to amino acid and nutrient status [53]. It has been suggested that this nutrient effect on mTOR might act through AMPK, as activation of this kinase is reduced when Ser^2448^ is phosphorylated. Interestingly, Ser2448 phosphorylation was increased in placentas from APNtg dams, and placentas from APNtg fetuses had an even higher phosphorylation, suggesting that adiponectin can modulate mTOR activity by increasing Ser2448 phosphorylation. Moreover, insulin receptor substrate 2 (IRS2) acts downstream of the insulin‐like growth factor receptor (IGF‐R) and insulin receptor and is necessary for recruiting mTORC2. This positions IRS2 as a link between mTOR and IGF/insulin signaling pathways [54]. In our study, we identified three differentially phosphorylated sites in IRS2: Thr517, Thr524, and Ser556, in placentas from adiponectin overexpressing dams. These findings suggest that adiponectin may regulate mTOR through IRS2 phosphorylation, potentially affecting IGF transport and uptake. Placental mTOR signaling also influences the metabolic function of fetal tissues by regulating IGF bioavailability [55]. Inhibition of mTOR in decidual cells has been linked to increased expression of IGF‐binding proteins (IGFBPs), leading to decreased free IGF, commonly observed in growth restricted fetuses [5]. Similarly, elevated maternal adiponectin in this study was associated with reduced fetal growth and the enrichment of pathways related to the regulation of IGF transport and uptake by IGFBPs.

Adiponectin also decreased the uptake of glucose and fatty acids. Previous studies have shown that adiponectin decreases placenta glucose uptake via decreased insulin signaling [19, 56], while the reduced fatty acid uptake is a new finding. The proportion of fatty acids on the maternal side of the syncytiotrophoblast that are transferred to the fetal circulation is largely unknown, although some information comes from a human in vivo study using fatty acid isotopes that estimate fatty acid at 0.5% [57]. Interestingly, about 0.5% of the glucose and fatty acid tracers administered to wt dams were enriched in placenta and fetal liver, while this was about half in APNtg dams. In the placenta, fatty acids are either used for mitochondrial oxidation, lipid droplet formation, or transported to the fetus [52]. Storage as lipid droplets was unaltered as triglycerides in the placenta were similar between groups. Lipoprotein lipase (LPL) needs to hydrolyze triglycerides in the maternal circulation to make fatty acids available for uptake into the placenta through fatty acid transport proteins. The increased LPL expression in placentas from obese APNtg dams may reflect a compensatory mechanism for the decreased fatty acid uptake seen in vivo, or compensation for a possible reduced LPL activity, which has been observed in intrauterine growth‐restricted placentas [58].

Excessive activation of the complement system is likely involved in placental dysfunction and pregnancy complications such as preeclampsia, as preeclamptic mouse models are associated with deposition of complement component 3 (C3) in placentas [59]. Proteins involved in the complement cascade, including C3, were upregulated in placentas from obese APNtg dams. Adipsin and C3 are increased in absorbed placentas from mice and may play a role in the maternal‐fetal interface [47]. Although adipsin and C3 were more highly expressed in the placentas of APNtg mice, the rate of resorption was not increased, which may have been rescued by the anti‐inflammatory effects of adiponectin, which are thought to downregulate immune system processes.

### 4.2 Adiponectin and Liver Function

Fetal liver function plays a key role in the regulation of fetal growth, as the umbilical vein enters the liver first before nutrients enter the systemic circulation. Tracer uptake in the placenta was mirrored in the fetal livers, and the decreased glucose, amino acid, and fatty acid uptake triggered the downregulation of metabolic pathways in the liver of APNtg fetuses. Reduced amino acid availability can impair protein synthesis, restricting fetal growth and organ development. The liver proteome indicates a starvation response with lower expression of proteins regulating amino acid metabolism and fatty acid oxidation, and the accumulation of liver triglycerides in fetuses from APNtg dams indicates an overall slower metabolism. Both AMPK and PPAR signaling pathways were downregulated in the fetal livers from APNtg dams, which could explain the decrease in fatty acid oxidation and the accumulation of triglycerides in the fetal liver. A placenta with reduced nutrient transport capacity sets off a cascade of compensatory metabolic changes in the fetal liver, which can have immediate but also long‐term consequences via epigenetic modifications in key metabolic genes. It remains to be determined whether the fetal liver changes have a negative impact on the metabolic functions in the adult offspring or whether the imprinting effects of maternal adiponectin can protect against these effects [21, 27].

### 4.3 Translational Aspects

Total adiponectin concentrations in the umbilical cord blood increase with gestational age and are 4–7 fold higher than those in the mother's blood at delivery [25], and are likely produced by the baby. Mouse fetuses also produce adiponectin during late pregnancy as shown here and by others [17, 18, 60]. However, there is a big difference between human and mouse fetuses in fat deposition. Fat deposition increases exponentially during the last weeks of pregnancy in babies while mice are born largely without white adipose tissue [61]. Fetal adiponectin is produced by brown adipose tissue in mice and may program the recruitment of mesenchymal stem cells [41]. A mouse embryo at GD 18 is roughly equivalent in developmental stage to a human fetus at about 22–24 weeks of gestation based on the relative maturity of organ systems and the overall physical development of the fetus. It is important to consider this equivalence when using mouse models to study aspects of human development and interpret experimental results in the context of human biology.

### 4.4 Conclusion

There is still no consensus on the effects of pregestational obesity and maternal and neonatal adiponectin levels on growth trajectories and childhood adiposity. We conclude that elevated maternal adiponectin in obese dams leads to decreased placenta nutrient transport, fetal growth restriction, and altered fetal liver function. Maternal adiponectin levels were the main determinant of placenta function, and our results suggest that high maternal adiponectin in combination with obesity during pregnancy may be detrimental to the offspring. It remains to be determined whether growth restriction has a negative effect on the adult offspring or whether the programming effects of adiponectin on metabolic functions compensate for any negative intrauterine effects.

## Author Contributions

A.B., E.S.‐V., and I.W.A. designed the study and interpreted data. M.S., B.U., S.S.S., M.B.T., M.M.S., C.O., and M.M. performed research, analyzed, and interpreted data. A.B. and M.S. drafted the manuscript. All authors reviewed/edited the manuscript and approved the final version of this manuscript.

## Conflicts of Interest

The authors declare no conflicts of interest.

## Supporting information


Table S1.

Table S2.

Table S3.

Table S4.

Table S5.

Table S6.

Table S7.

Table S8.

Table S9.

Table S10.

Table S11.

Figure S1.

Figure S2.

Figure S3.

## Data Availability

The data supporting the findings of this study are available on request with the corresponding author. The mass spectrometry proteomics data have been deposited to the ProteomeXchange Consortium via the PRIDE partner repository with the dataset identifier PXD025390.
