# Supplementary material for: Maternal Adiponectin Decreases Placenta Nutrient Transport in Mice
Source: FASEB J. 2025 Apr 18;39(8):e70556. doi: 10.1096/fj.202403251RR (PMC12007623; doi:10.1096/fj.202403251RR)
Supplement: Supplementary file 12 — Figure S1. [file FSB2-39-e70556-s007.pptx]

## Slide 1
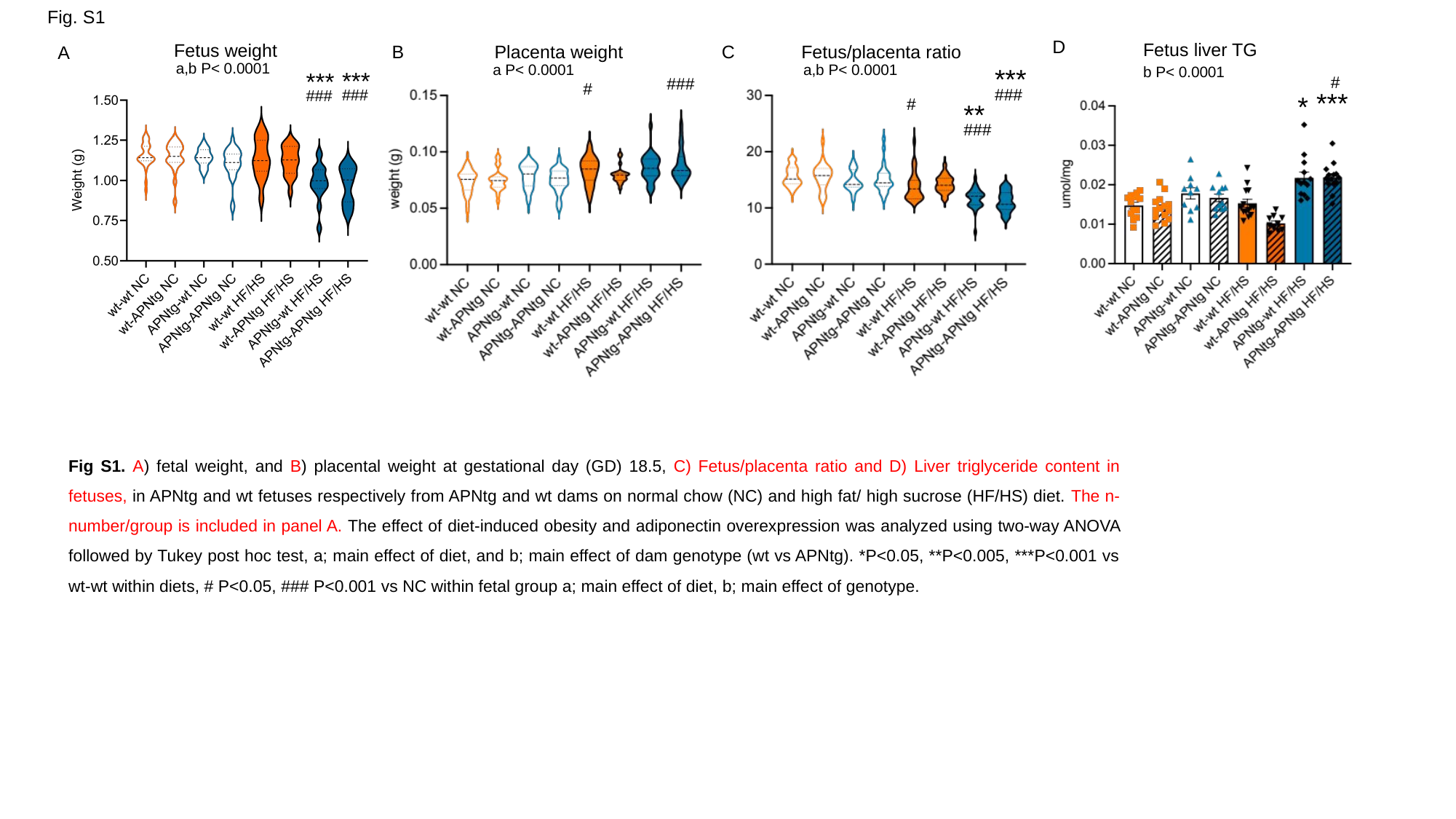

Fig. S1
D
Fetus liver TG
Fetus weight
B
Placenta weight
C
Fetus/placenta ratio
A
a,b P< 0.0001
a P< 0.0001
a,b P< 0.0001
b P< 0.0001
#
###
***
###
***
###
***
###
#
*
#
***
**
###
Fig S1. A) fetal weight, and B) placental weight at gestational day (GD) 18.5, C) Fetus/placenta ratio and D) Liver triglyceride content in fetuses, in APNtg and wt fetuses respectively from APNtg and wt dams on normal chow (NC) and high fat/ high sucrose (HF/HS) diet. The n-number/group is included in panel A. The effect of diet-induced obesity and adiponectin overexpression was analyzed using two-way ANOVA followed by Tukey post hoc test, a; main effect of diet, and b; main effect of dam genotype (wt vs APNtg). *P<0.05, **P<0.005, ***P<0.001 vs wt-wt within diets, # P<0.05, ### P<0.001 vs NC within fetal group a; main effect of diet, b; main effect of genotype.
