# Supplementary material for: Maternal Adiponectin Decreases Placenta Nutrient Transport in Mice
Source: FASEB J. 2025 Apr 18;39(8):e70556. doi: 10.1096/fj.202403251RR (PMC12007623; doi:10.1096/fj.202403251RR)
Supplement: Supplementary file 13 — Figure S2. [file FSB2-39-e70556-s002.pptx]

## Slide 1
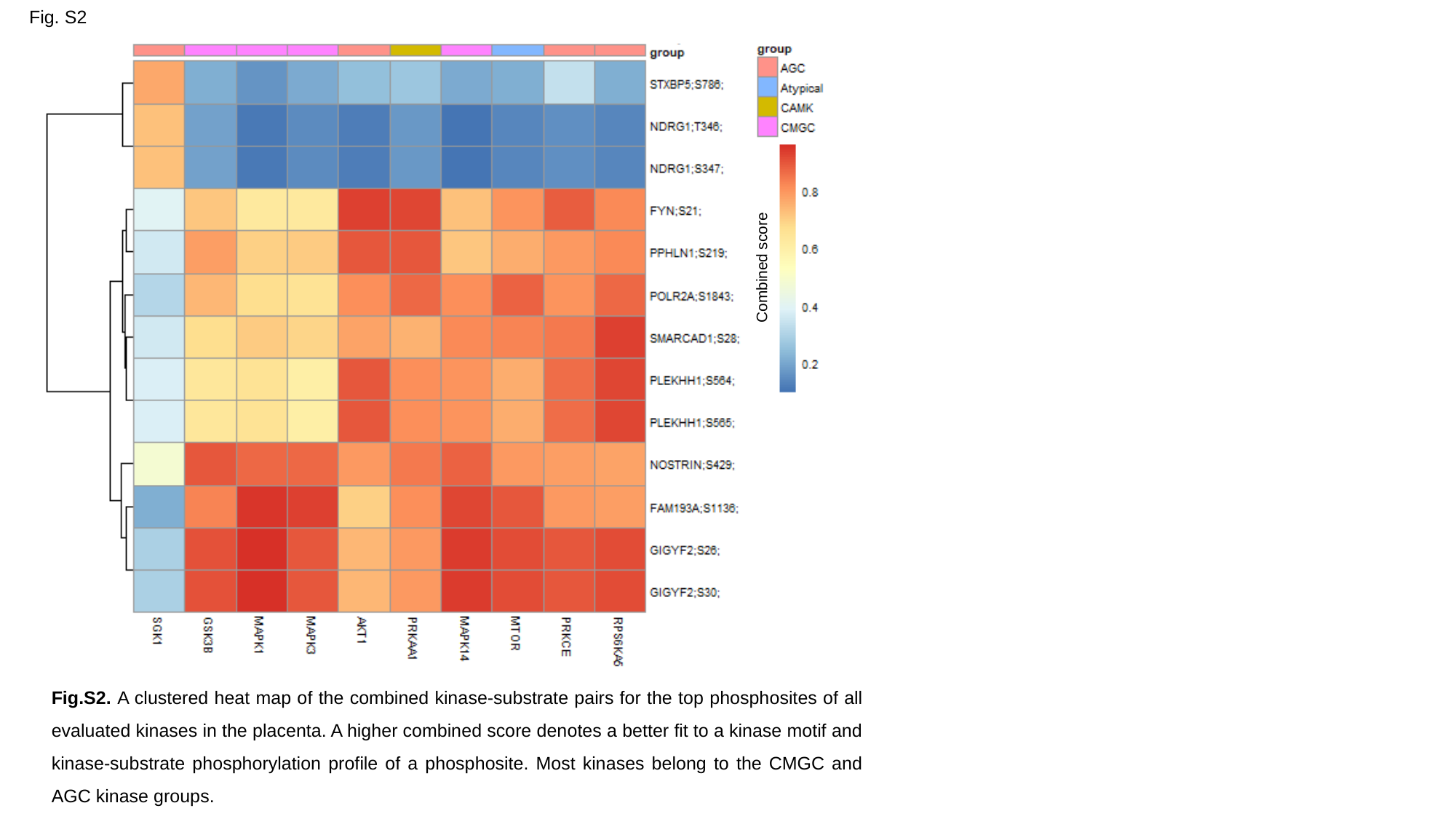

Fig. S2
Combined score
Fig.S2. A clustered heat map of the combined kinase-substrate pairs for the top phosphosites of all evaluated kinases in the placenta. A higher combined score denotes a better fit to a kinase motif and kinase-substrate phosphorylation profile of a phosphosite. Most kinases belong to the CMGC and AGC kinase groups.
