# Supplementary material for: Maternal Adiponectin Decreases Placenta Nutrient Transport in Mice
Source: FASEB J. 2025 Apr 18;39(8):e70556. doi: 10.1096/fj.202403251RR (PMC12007623; doi:10.1096/fj.202403251RR)
Supplement: Supplementary file 14 — Figure S3. [file FSB2-39-e70556-s009.pptx]

## Slide 1
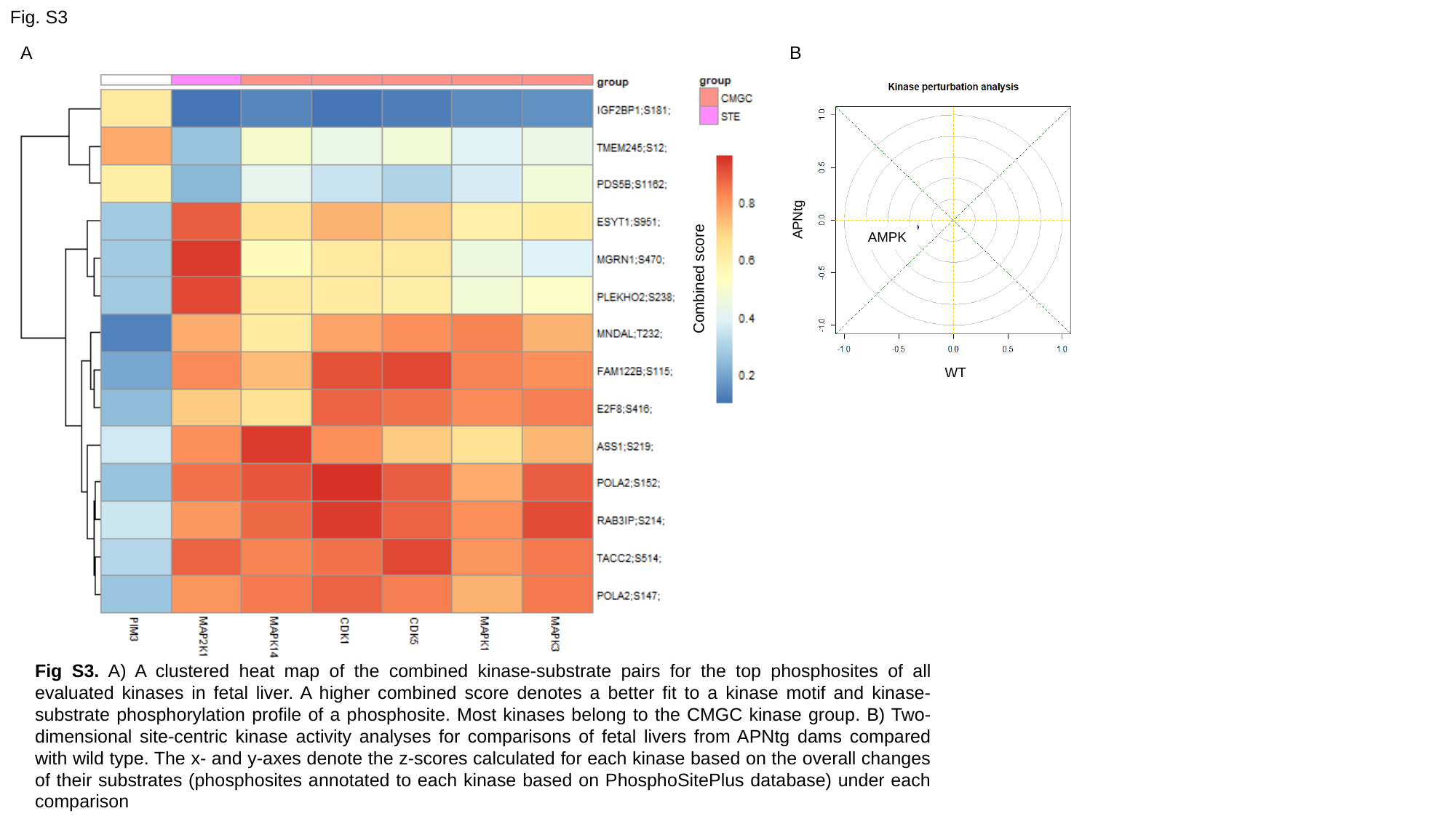

Fig. S3
B
A
APNtg
AMPK
WT
Combined score
Fig S3. A) A clustered heat map of the combined kinase-substrate pairs for the top phosphosites of all evaluated kinases in fetal liver. A higher combined score denotes a better fit to a kinase motif and kinase-substrate phosphorylation profile of a phosphosite. Most kinases belong to the CMGC kinase group. B) Two-dimensional site-centric kinase activity analyses for comparisons of fetal livers from APNtg dams compared with wild type. The x- and y-axes denote the z-scores calculated for each kinase based on the overall changes of their substrates (phosphosites annotated to each kinase based on PhosphoSitePlus database) under each comparison
